# Synthesis, Structural Characterization and Antimicrobial Activity of Cu(II) and Fe(III) Complexes Incorporating Azo-Azomethine Ligand

**DOI:** 10.3390/molecules23040813

**Published:** 2018-04-02

**Authors:** Mohammad Azam, Saud I. Al-Resayes, Saikh Mohammad Wabaidur, Mohammad Altaf, Bhaskar Chaurasia, Mahboob Alam, Satyendra Nath Shukla, Pratiksha Gaur, Nader Talmas M. Albaqami, Mohammad Shahidul Islam, Soonheum Park

**Affiliations:** 1 Department of Chemistry, College of Science, King Saud University, P.O. Box 2455, Riyadh 11451, Saudi Arabia; sresayes@ksu.edu.sa (S.I.A.-R.); tarabai22@gmail.com (S.M.W.); altafamu@gmail.com (M.A.); nader___t@hotmail.com (N.T.M.A.); shahid10amui@gmail.com (M.S.I.); 2 Coordination Chemistry Research Laboratory, Department of Chemistry, Govt. Science College, Jabalpur 482001, India; bc3088@gmail.com (B.C.); ccrl_2004@rediffmail.com (S.N.S.); drgaur26@gmail.com (P.G.); 3 Division of Chemistry and Biotechnology, Dongguk University, 123 Dongdae-ro, Gyeongju 780-714, Korea; mahboobchem@gmail.com; 4 Department of Advanced Materials Chemistry, Dongguk University, 123 Dongdae-ro, Gyeongju 780-714, Korea; shpark@dongguk.ac.kr

**Keywords:** azo ligand, synthesis, DFT studies

## Abstract

We are reporting a novel azo-azomethine ligand, HL and its complexes with Cu(II) and Fe(III) ions. The ligand and its complexes are characterized by various physico-chemical techniques using C,H,N analyses, FT-IR, ^1^H-NMR, ESI-MS and UV-Vis studies. TGA analyses reveal complexes are sufficiently stable and undergo two-step degradation processes. The redox behavior of the complexes was evaluated by cyclic voltammetry. Furthermore, the ligand and its complexes were tested for antimicrobial activity against bacterial and fungal strains by determining inhibition zone, minimal inhibitory concentration (MIC) and minimal bactericidal concentration (MBC). The complexes showed moderate antimicrobial activity when tested against Gram +ve and Gram −ve bacterial strains. To obtain insights into the structure of ligand, DFT studies are recorded. The results obtained are quite close to the experimental results. In addition, the energy gap, chemical hardness, softness, electronegativity, electrophilic index and chemical potential were calculated using HOMO, LUMO energy value of ligand.

## 1. Introduction

Over the years, the azo compounds with at least one azo (–N=N–) group, separated by two phenyl rings, have received tremendous attention both in fundamental and applied research [1,2,3,4,5,6]. Furthermore, the azo compounds are used extensively in textile industry, printing systems, biological staining and various photochemical productions [7,8,9]. In addition, the azo-Schiff bases are reported to display various antimicrobial, anticancer and antioxidant activities, and several other pharmacological properties [3,10,11,12,13,14].

Over the last few decades, several antibacterial and antifungal drugs, such aspenicillin, cephalosporins, tetracyclines, macrolides and oxaolidinones, fluconazole, ketoconazole and miconazole are being explored to treat the infection caused by various microorganisms [15]. However, there are reports stating that bacteria release specific things out of the cell, thus making the access of antibiotic to the active site limited, and prevents the accumulation of antibiotic in the interior of the cell, and thus inhibit the action of antimicrobial drug [16]. For example, the β-lactam antibiotic inhibits penicillin-binding proteins (PBPs), an enzyme required for the biosynthesis of bacterial cell wall, is now suffering from resistance due to the release of β-lactamases enzyme, making the drug inactive [17,18]. Similarly, significant occurrence of methicillin-resistant *S. aureus* (MRSA) is steadily rising [19,20,21]. In last two decades, several new antibiotics failed to meet the challenges posed by multidrug-resistant pathogens [19,20,21]. Therefore, we need such drugs which are sufficient to counteract and control the antibiotic resistance [19,20,21]. Over the years, various metal containing compounds have received significant attention in various biological applications, especially anticancer and antimalarial therapy. However, less attention has been given to develop the metal containing antibacterial drugs [20,22]. Therefore, investigations for metal-based antibacterial compounds are required as it anticipated that metal-based drugs may be helpful to overcome the development of antibiotic resistance [20].

Herein this article, we are concerned with the synthesis of a novel azo-azomethine ligand, 2-((E)-(pyridin-2-ylimino)methyl)-4-((E)-p-tolyldiazenyl)phenol and its complexes with Cu(II) ion and Fe(III) ions. The structure of ligand and its complexes have been confirmed by elemental analyses, FTIR, NMR ESI-MS, UV/Vis, thermal and electrochemical studies. In addition, DFT studies have been carried to look insights the bonding of the ligand. Furthermore, the ligand and its complexes showed potential antimicrobial activity when screened against Gram +ve and Gram −ve pathogens. 

## 2. Results and Discussion

**Scheme 1:**
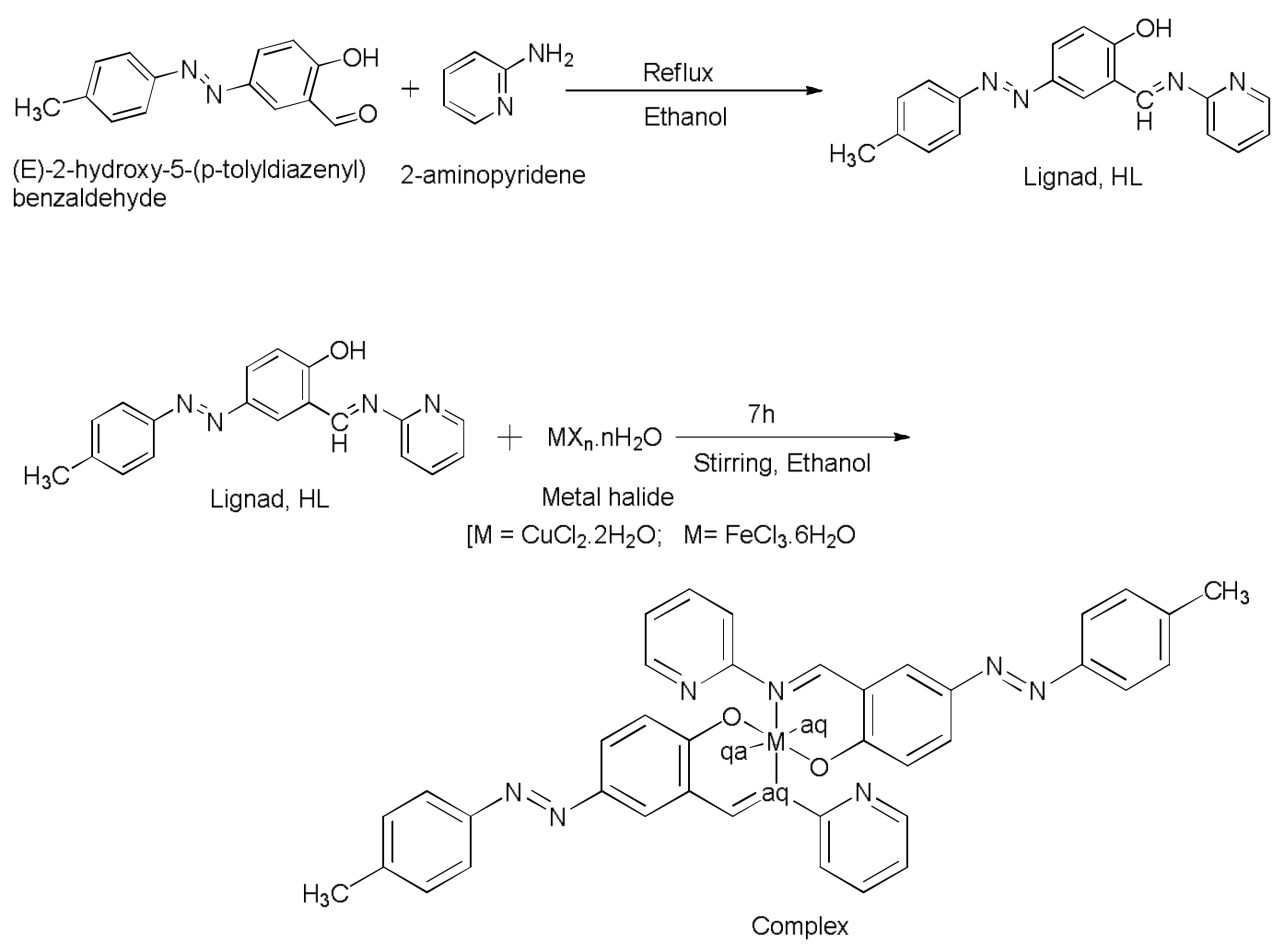
Preparation of azo-azomethine ligand, HL and its complexes with Cu(II) and Fe(III) ions.

The synthesis of ligand, HL and its complexes is described in Scheme 1. The mass spectral studies of the azo-Schiff base ligand, HL and its complexes 1 and 2 were recorded by the electron impact mass spectrum and its data are given in experimental section. The molecular ion peak [M + H]^+^ *m/z* at 316.1, 731.2 and 723.5 are corresponding to their molecular formulae, C_19_H_16_N_4_O, C_38_H_34_CuN_8_O_4_and C_38_H_34_FeN_8_O_4_, respectively. On the other hand, the calculated *m*/*z* for ligand, HL and its complexes 1 and 2 were found to be 316.4, 730.2 and 722.2, respectively (Supplementary Materials Figure S1). The stoichiometric analyses of ligand, HL and its complexes agree well with their proposed composition.

**Figure 1:**
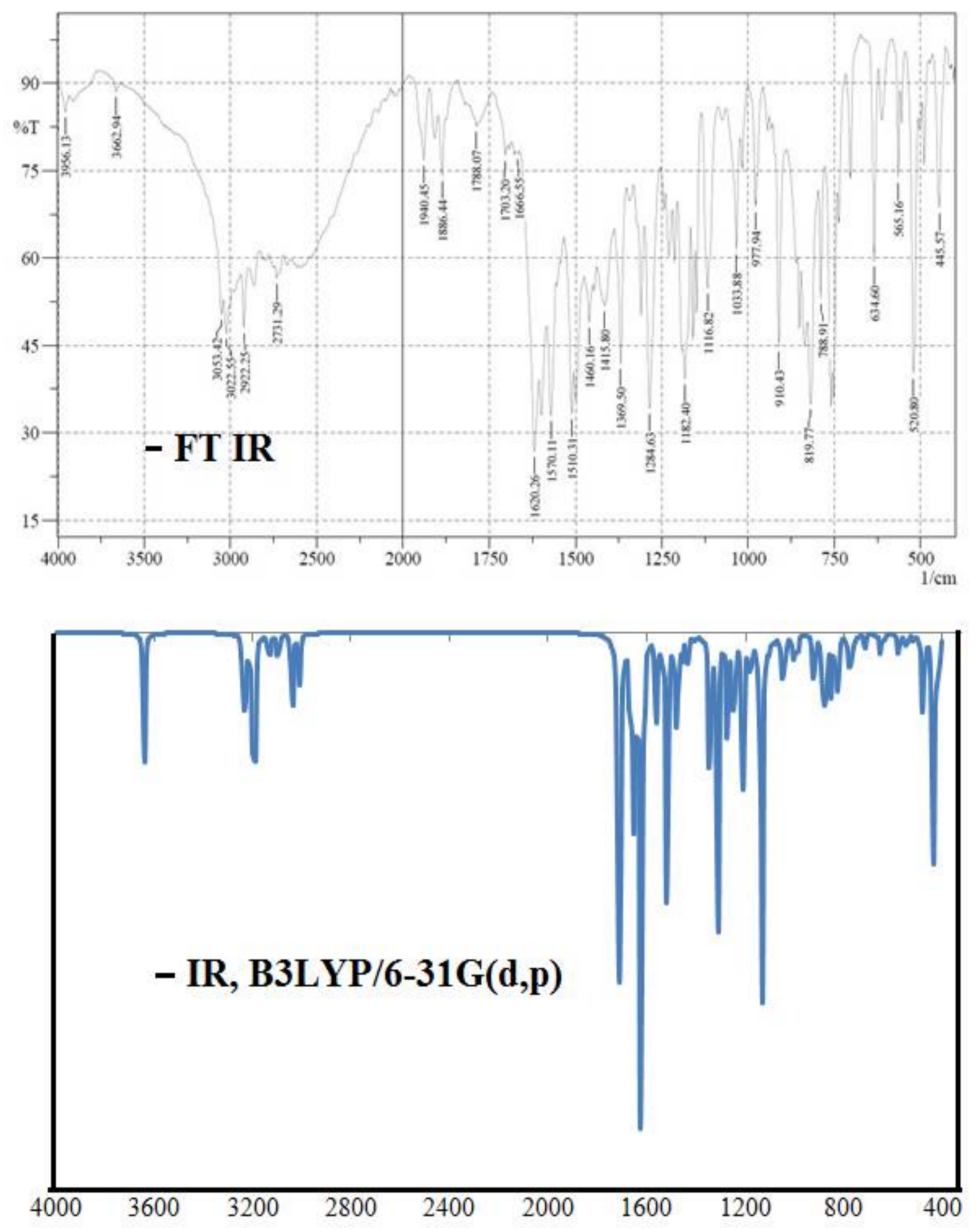
Comparison of experimental and simulated IR spectra of ligand, HL.

IR spectra of ligand, HL exhibited prominent peaks at 3053 cm^−1^, 1666 cm^−1^, 1460 cm^−1^ ascribed to ν_(–OH),_ ν_(–CH=N)_ and ν_(N=N)_ vibrations, respectively [23,24,25,26]. In addition, the stretching vibration due to ν_(C–O)_ band appeared at 1284 cm^−1^ in the free azo-Schiff base ligand [23,24]. However, position of these vibrations shifted from their original values upon complexation to metal ions [3,25,26,27]. In addition, the strong confirmation for the coordination of metal ion to ligand came from the absence phenolic OH group in the complexes 1 and 2 complexes showing that the coordination took place via the deprotonated OH group of the free azo-Schiff base ligand [3]. However, there was no change noticed in the position of –N=N– (azo group) in the complexes indicating that it did not take part in complex formation [23]. The IR values obtained from the simulated spectrum practically coincide with the experimental results, and are supported by the data reported in literature (Figure 1) [28].

**Figure 2:**
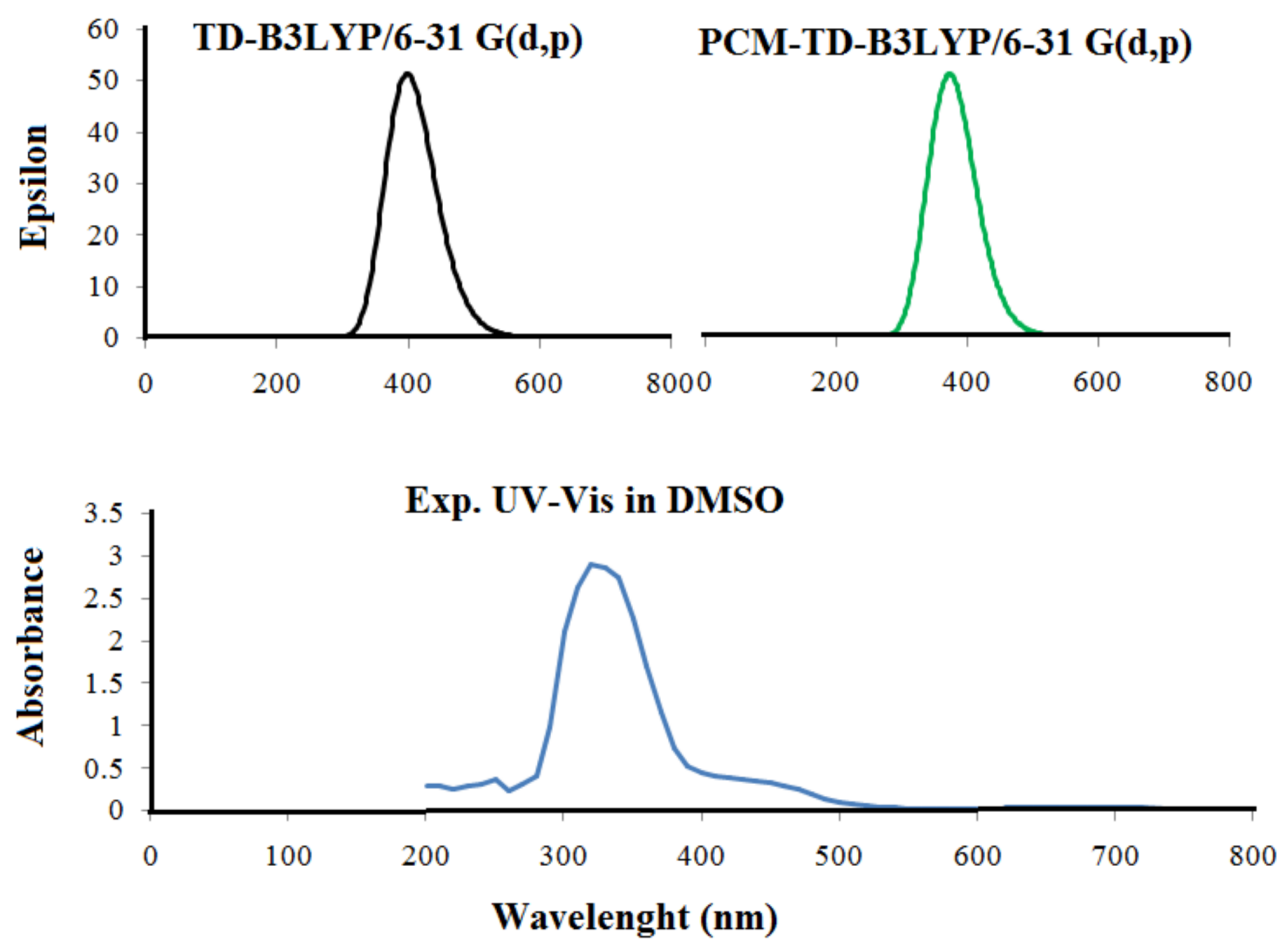
Comparative experimental and theoretical UV/Vis spectra.

The electronic spectrum of ligand (Figure 2) showed absorption bands at λ_max_250 nm and λ_max_320 nm due to low energy π-π* transitions [24,29].

To compare the experimental results, theoretical calculation were carried out in the solution as well as in the gaseous state by employing TD-DFT/B3LYP/6-31G(d,p) level of theory for the optimized structure (Figure 2). The observed absorption bands at 320 nm and 250 nm of the ligand in solution are assigned to various HOMO → LUMO transitions. However, the theoretical absorption bands are observed at 380 nm and 345 nm in solution and 402 and 380 nm in vacuum, suggesting that absorption spectrum in solution is closest to the experimental results. These values indicate that the UV in solution is more suitable than the gas phase for studying the absorption spectra of the ligand. The absorption bands are slightly changed to the higher wavelength due to solvent effect.

However, upon complexation, the position of absorption bands is shifted [30]. Furthermore, broad-band at λ_max_390 nm is assigned to n-π* transitions of –N=N– chromophore groups [24]. However, there was no significant change in these absorption bands in the complexes. The complex 1 exhibited a broad absorption band at 615 nm assigned to ^2^B_1g_ → ^2^B_2g_ transition indicating an octahedral geometry around Cu(II) ion [31,32], which is further confirmed by the magnetic susceptibility data at 1.7 BM [30]. The complex 2 showed absorption bands at340 nm and 370 nm corresponding to L → M charge transitions [33]. In addition, absorption bands at 440 nm and 510 nm were also observed, and assigned to ^6^A_1_g → ^4^T_1g_ and ^6^A_1g_ → ^4^T_2g_and ^2^T_2_ → ^2^A_2_ transition. Moreover, the magnetic moment value at 5.90 B.M suggests an octahedral environment around Fe (III) ion [32] (Supplementary Information Figure S2).

### 2.1. Frontier Molecular Orbitals—HOMO and LUMO of Ligand

**Figure 3:**
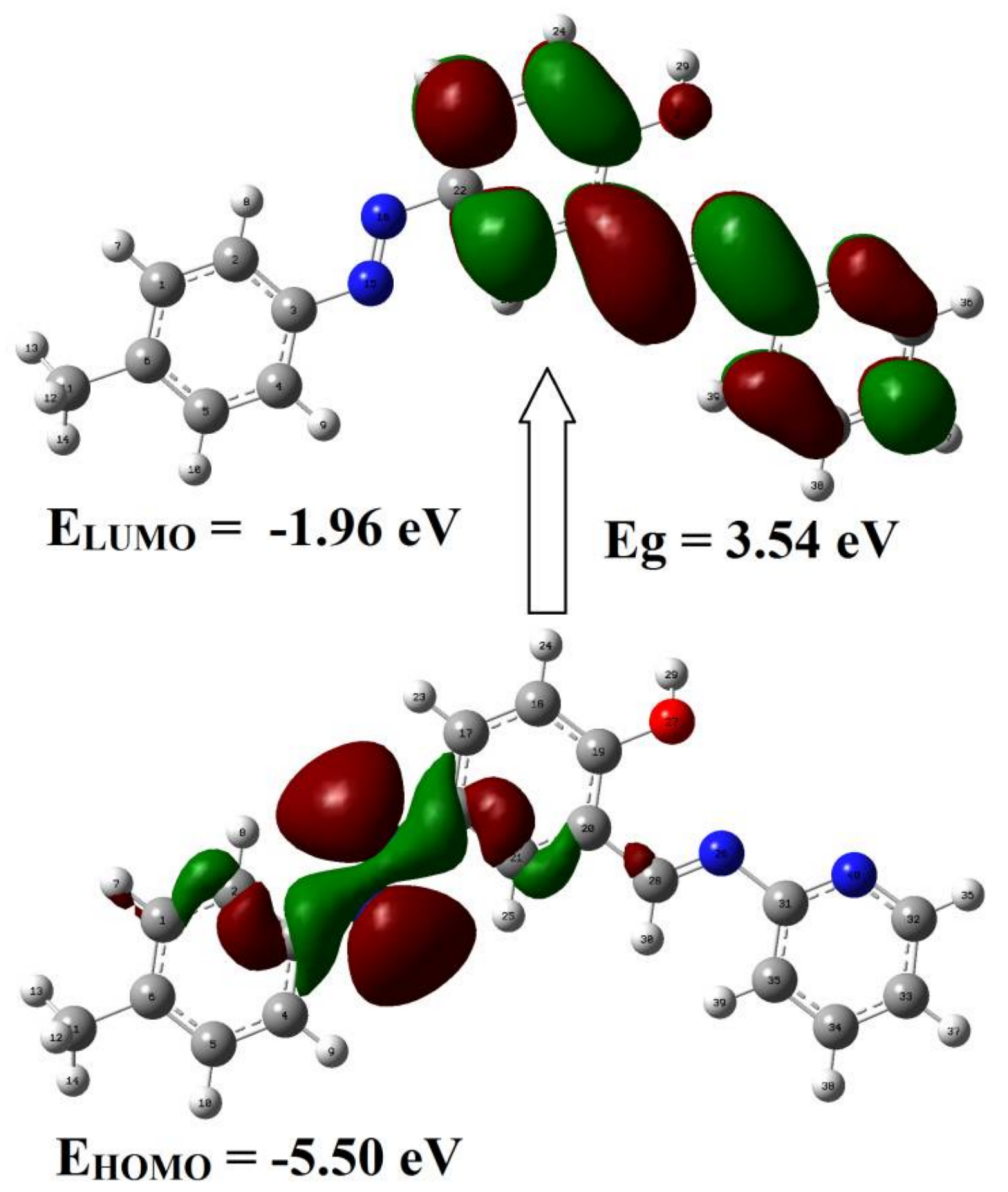
HOMO, LUMO and energy difference of ligand.

The study of molecular orbitals can bring useful information on the electronic framework and is widely applied in the analysis of chemical reactions [34]. Frontier molecular orbital (FMOs) energies were determined using the B3LYP/6-31G (d,p) level for the optimized molecular structure. The graphical presentation of HOMO, LUMO orbitals and Energy gap (Eg) are shown in Figure 3. The HOMO is largely located on diazo group around azomethine moiety and pyridine N, whereas LUMO is strictly confined over toluidine and phenolic ring. The HOMO-LUMO gap defines the chemical stability, reactivity and electron conductivity. The HOMO-LUMO gap for the ligand is 3.54 eV, suggesting its high reactivity towards bonding with metals. Furthermore, the HOMO energy shows that molecule is susceptible towards the electrophiles, whereas the LUMO energy indicates the susceptibility of the molecule towards the nucleophiles [35]. The chemical reactivity based on various quantum chemical parameters is given in Table 1.

### 2.2. Mulliken Charge Analysis of Ligand

**Figure 4:**
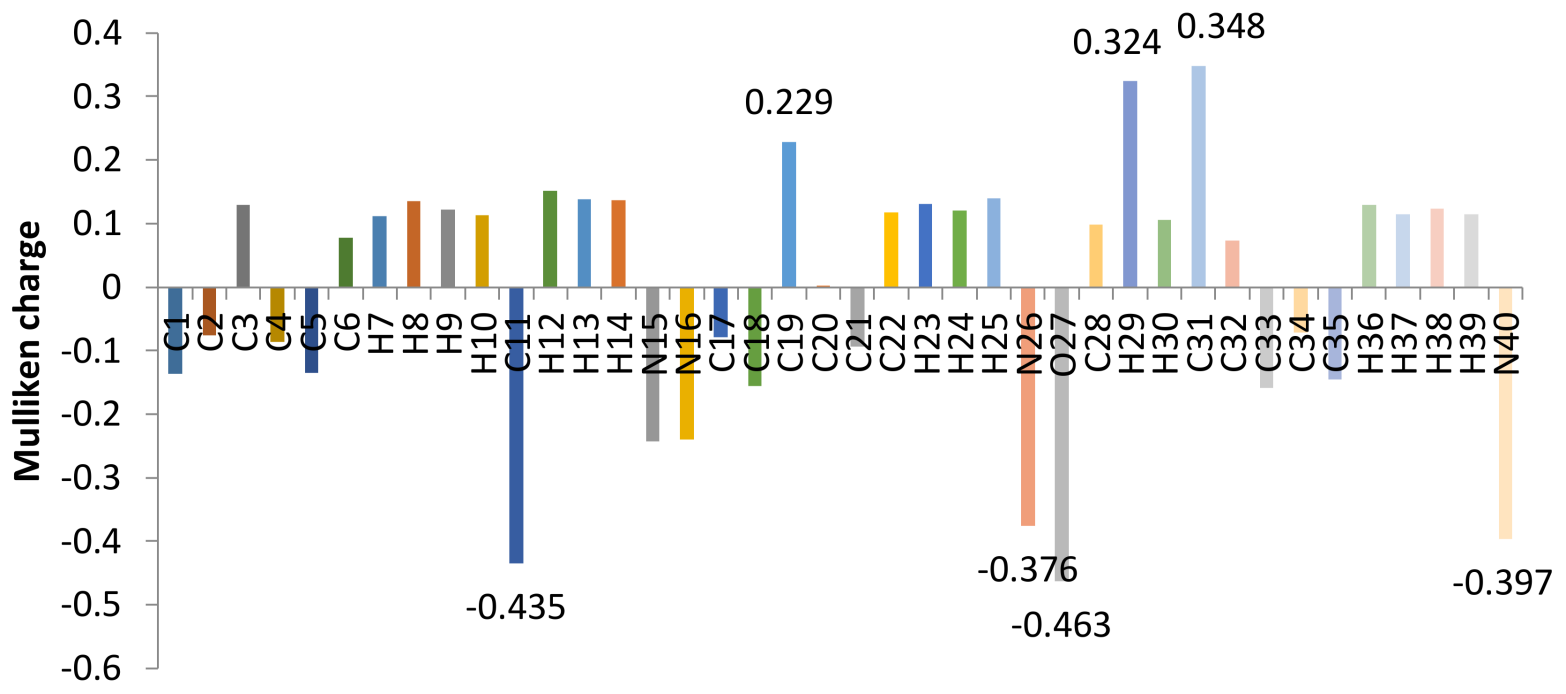
Mulliken Charge Distribution of ligand, HL.

The net atomic charges of the ligand have been described using Mulliken population analysis using B3LYP/6-31G basis set, and have a significant influence on vibrational spectra, dipole moment, molecular polarizability, electron structure and many properties of the molecular skeleton (Figure 4). In the ligand, the more positive charge on C31 + 0.348 carbon atom was due to the fact that this C31 carbon was flanked between two highly electro negative nitrogen, which was caused by the −I effect of nitrogen of azomethine N and Pyridine N atoms. Similarly C19 and H29 have positive charge of +0.324 and +0.329 respectively, due to their attachment with a strongly electronegative O atom. Highly shielded atoms such oxygen and nitrogen attached to less electronegative acquired highly negative charge, phenolic O27 and azomethine N26 being highly shielded having negative charge −0.376 and −0.463 respectively and pyridine N has a charge of −0.397 on the molecular system of the ligand.

### 2.3. Molecular Geometry of Ligand

**Figure 5:**
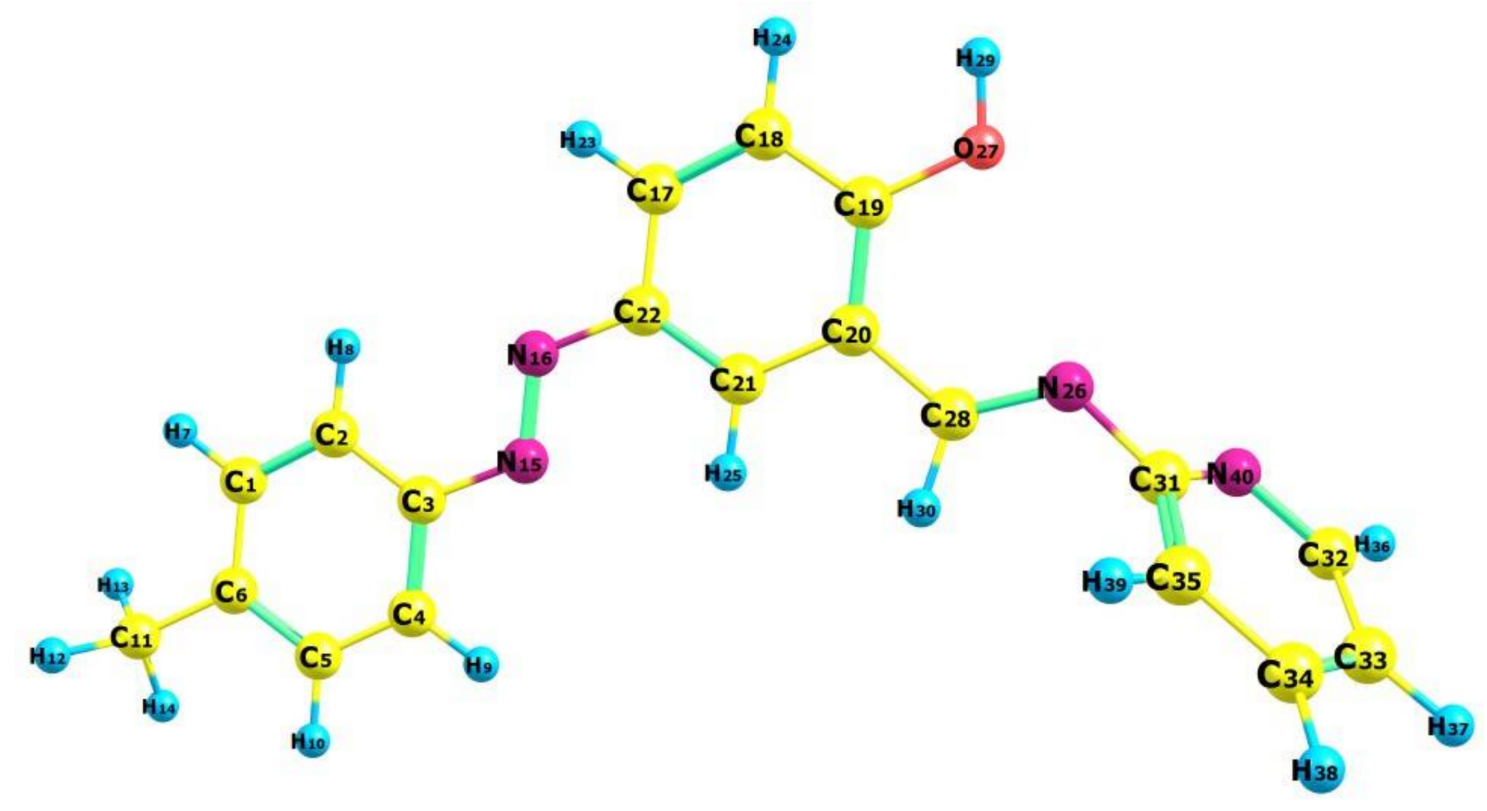
Optimized geometry of the ligand visualized using Chemcraft program.

The optimized ligand structure is non-planar and as shown in Figure 5 with the numbering scheme of the atoms. The optimized structural parameters given in Table 2 are comparable to XRD data with the similar structure [29]. It is reported that the optimized structural parameters are slightly different from the experimental results. However, 7° bond angle deviation has been reported for N15-N16-C3 in theoretical and experimental results [36]. Such deviations are likely due to inter/intra-interaction in crystal, whereas the theoretical studies are calculated for single isolated molecule without crystallographic information file (CIF) in vacuum. The minimum energy of the self-consistent field (SCF) of the molecule is −644,785.8000 kcal/mol in vacuum and −644,803.2394 kcal/mol in the solvent confirming that the geometry of the molecule is more stable in solvent phase because of the presence of hydrogen bonds. In addition, the optimized ligand geometry was also validated by frequency calculations, and provided real values for the frequencies obtained, and no imaginary frequency was found. Positive values indicate that the structure has minimal potential energy. Therefore, we can propose an optimized structure (Figure 5) for the ligand on the basis of elemental analyses, FT-IR, Electronic spectra, ^1^H-NMR, and DFT study.

**Figure 6:**
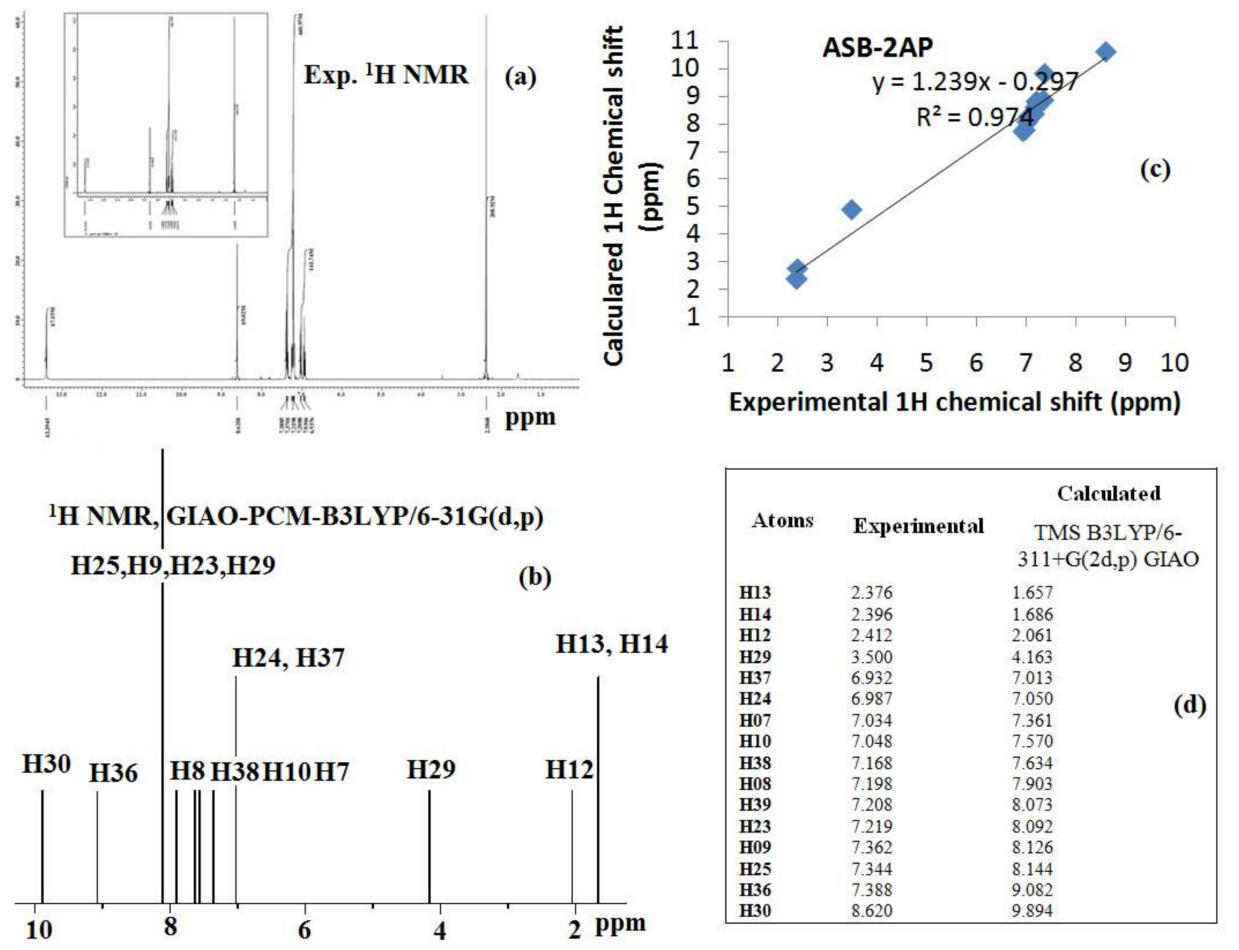
Comparison of (**a**) experimental and (**b**) theoretical ^1^H-NMR spectra (**c**) correlation graph and (**d**) experimental and calculated ^1^H chemical shifts (ppm) of ligand, HL.

^1^H-NMR spectrum of ligand, HL (Figure 6) displayed a set of signals in the range of δ 0–14 ppm corresponding to various positions within the ligand. The singlet due to methyl protons appear at δ 2.20 ppm. The signal is appeared as two doublets at δ 6.93 and δ 7.03 ppm were assigned for toluidine aromatic protons. Similarly the singlet at δ 7.21 for 1H and two doublets at δ 7.20 and δ 7.18 for 1H each, were attributed for all the three protons of phenolic ring. The signal appeared at δ 7.34, δ 7.37 and δ 7.38 ppm were assigned for protons of pyridine ring. A signal at δ 8.62 ppm for 1H was assigned for one azomethine (–CH=N–) proton. Phenolic OH generally resonates at very high frequency in the *deshielding region* of the NMR spectrum. In our study, the ligand shows the phenolic proton as a singlet at δ 13.39 ppm (s, 1H, OH). The ^1^H-NMR spectrum of ligand has been compared with the experimental data and is given in Figure 6a–d. The calculation of ^1^H-NMR spectrum was carried out in dimethyl sulfoxide (DMSO) solution using PCM model within GIAO-B3LYP framework. However, the expected NMR chemical shifts were not fully compatible with the experiment results. As can be seen in Figure 6c, some of the peaks such as hydroxyl and methyl protons appeared in spectrum were deviated from standard values, which can be explained on the basis of steric hindrance, solvent effect, and stereochemistry. Furthermore, the effects of the solvent were found for the OH proton to a varying extent. The correlation coefficients of ^1^H-NMR for ligand were determined to be 0.974 as shown in Figure 6c. (Ligand^1^Hδ_cal_ = 1.239δ_exp_ − 0.297 (R^2^ = 0.974)). Predicted chemical shift values applying linear regression were found to be in reasonable agreement with the experimental values.

### 2.4. Thermal Studies

**Figure 7:**
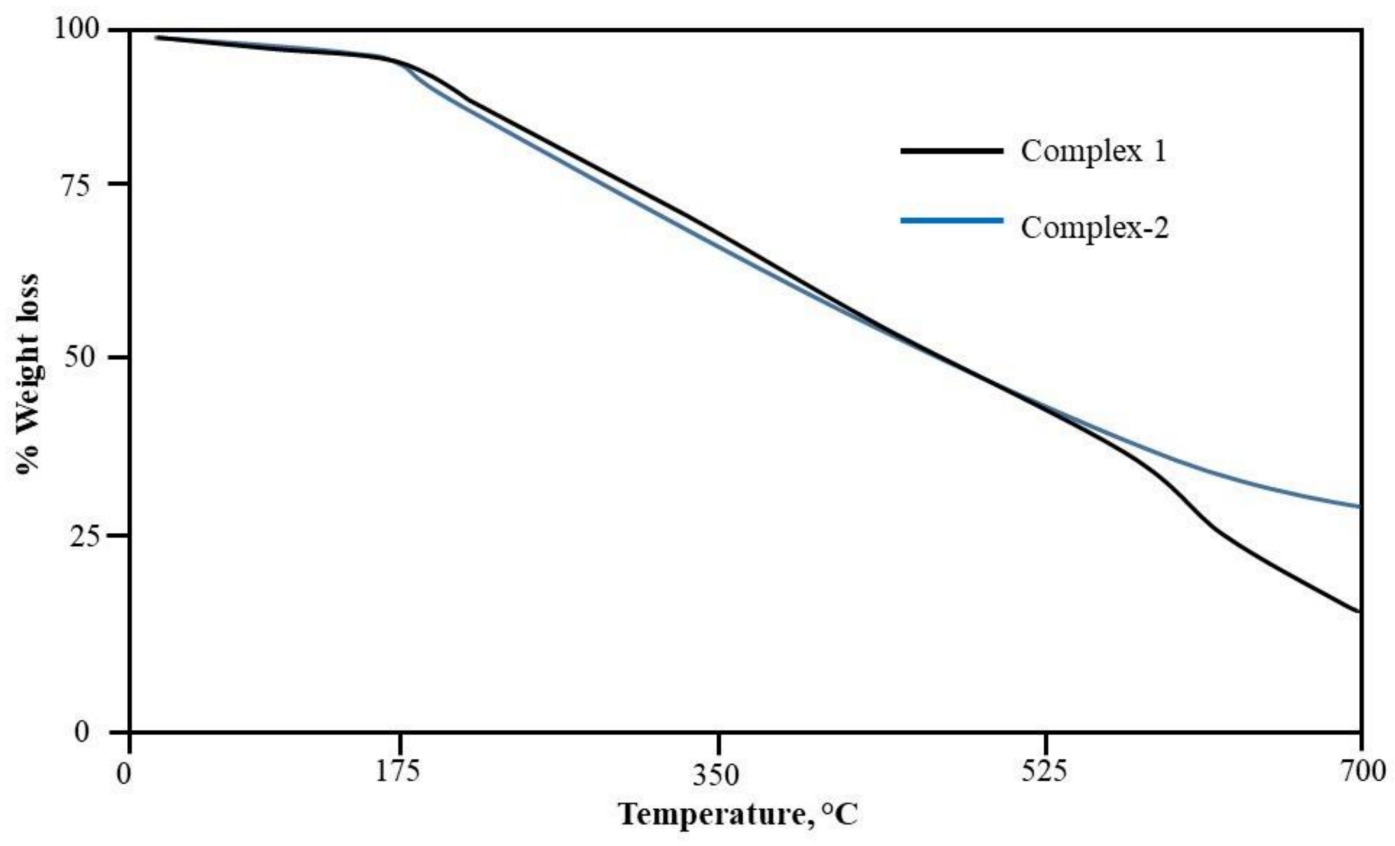
TGA curve of complexes 1 and 2.

TGA results (Figure 7) show that both the complexes degrade almost in same way, and the whole degradation occurs in two steps. The first step in both complexes include the loss of both the coordinated water molecules at temperature 160–170 °C, which is attributed to 5.0% of the total weight loss in the complexes 1 and 2. Upon increasing temperature, complexes started degrading and complex 1 finally decomposed at temperature 600–630 °C, corresponding to weight loss (84.11%) leaving copper oxide in the end as end product. However, complex 2 is decomposed completely at temperature 650 °C, which is due to the loss of whole organic moiety (72.9% of the complex), leaving metal oxide in the end as end product.

The redox properties of complexes 1 and 2 (1.0 × 10^−3^ mol L^−1^) were investigated by cyclic voltammetry in the potential range +1.0 to −1.5 V in degassed DMSO solution in the presence tetrabutylammonium perchlorate (0.01 mol L^−1^) as the supporting electrolyte at a scan rate of 100 mVs^−1^. The cyclic voltammogram of complex 1 shows two cathodic peaks, observed at potential −1.39 V and −0.92 V and one anodic peak at 0.45 V. However, irreversible oxidation peak is found 0.65 V [37,38]. Complex 2 exhibited two reduction waves at 0.87 V and 0.58 V attributed to the reduction of Fe(III) to Fe(II) and reduction of azo ligand [39,40].

### 2.5. Antimicrobial Activity

The studied azo-azomethine ligand and its complexes 1 and 2 exhibited significant antimicrobial activity against bacterial strains. However, on comparing the results with the existing antibiotic and antifungal drugs, the ligand and its complexes showed moderate activity against these pathogens. However, ligand and complexes 1 and 2 showed insignificant effect over fungal strains. Interestingly, complexes 1 and 2 exhibited better activity as compared to the free ligand. The complex 1 showed high sensitivity against *E. coli* in comparison to *Streptococcus aureus* (MRSA) (Table 3). The MIC and MBC values obtained for ligand and its complexes against the bacterial and fungal strains are summarized in Table 3. The biological study data in the case of the antibacterial assay discloses that the complexes indicate antimicrobial activity higher than that of the ligand. Such improved activity can be explained on the basis of the theory of chelation.

## 3. Materials and Methods

Salicylaldehyde, 2-aminopyridine, CuCl_2_.2H_2_O, FeCl_3_.6H_2_O were obtained from E. Merck. (E)-2-hydroxy-5-(p-tolyldiazenyl)benzaldehyde used in the synthesis of azo-Schiff base ligand was obtained as reported in literature [2,37]. Electronic absorption spectra were measured using EI-2305 spectrometer. FT-IR spectra were recorded on Shimadzu-8400 PC FT-IR spectrophotometer. ^1^H-NMR spectra was carried out on Jeol spectrometer at 400 MHz with chemical shifts relative to TMS in d_6_-DMSO. The ESI-MS spectra were recorded on an Agilent 6520 (QTOF) mass spectrophotometer. The electrochemical behavior of the complexes was recorded on Ivium technologies potentiostat/galvanostat in a three-electrode cell with glassy carbon (GC) working electrode, a platinum wire auxiliary counter electrode, and Ag/AgCl as a reference electrode. Thermal stability was monitored on SDTQ-600 (TA Instrument) in helium (100 mL min^−1^) at 20 °C/min at 0–700 °C.

### 3.1. Synthesis of Ligand, (4E)-4-(2-p-tolyldiazenyl)-2-((E)-(pyridin-2-ylimino)methyl)phenol, HL

An ethanolic solution of (E)-2-hydroxy-5-(p-tolyldiazenyl)benzaldehyde (2.440 g, 10 mmol) was added gradually to the solution of 2-amino pyridine (0.941 g, 10 mmol) dissolved in minimum quantity of same solvent. The resulting reaction mixture was refluxed for 5 h, and allowed to cool at room temperature. A brown colored solid product was obtained, which was removed by filtration and washed with diethylether and dried in vacuum to obtain analytically pure compound.

Color: Brown, yield 68%, mp > 280 °C, molecular formula C_19_H_16_N_4_O; elemental analysis found: C, 72.02; H, 5.02; N, 17.65; Cal. C, 72.13; H, 5.10; N, 17.71%, FT-IR data (KBr, cm^−1^): 3053 ν_(–OH)_; 1666 ν_(–CH=N)_, 1460 ν_(–N=N–)_; ^1^H-NMR (DMSO-d_6_, 400 MHz, δ in ppm): 8.94 (s, 1H, –CH=N), 2.20 (s, 3H, –CH_3_), 13.39 (s, 1H, Ar-OH), 6.93–7.38 (m, 11H, Ar-H); electronic spectrum λ_max_(nm): 250, 320; ESI-mass spectra *m*/*z*: [C_19_H_16_N_4_O+H]^+^ 316.1.

### 3.2. Synthesis of Complexes

#### 3.2.1. Synthesis of Complex 1, [Cu(L_2_)(H_2_O)_2_]

CuCl_2_.2H_2_O (50 mg, 0.292 mmol) was added to the ethanolic solution of azo-Schiff base ligand, HL (185 mg) at room temperature. The resulting reaction mixture was stirred vigorously for 7 h. There was no change noticed in the color of reaction mixture and remains brown. The solution of reaction mixture was reduced under vacuum and precipitated out by adding diethyether, and recrystallized in ethanol. After few days, some brown colored microcsaline product was obtained. Unfortunately, we did not find any crystal suitable for single crystal XRD.

[Cu(L_2_)(H_2_O)_2_]: Yield 63.24%, Color: brown, molecular formula C_39_H_37_CuN_8_O_4_, elemental analysis found: C, 62.79; H, 4.91; N, 14.91; Cu, 9.02; Cal: C, 62.85; H, 5.00; N, 15.03; FT-IR data (KBr, cm^−1^): 1673 ν_(–CH=N)_, 1460 ν_(–N=N–)_; UV/Vis (λ_max_nm): 615; ESI-Mass spectra, *m*/*z*: [C_38_H_34_CuN_8_O_4_ + H]^+^ = 731.

#### 3.2.2. Synthesis of Complex 2, [Fe(L)_2_2H_2_O]

Complex 2 was synthesized in the same way as complex 1

[Fe(L)_2_(H_2_O)_2_]:Color: Reddish-brown, Yield 68.74%, mp, >300 °C, elemental analysis found: C, 63.08; H, 4.68; N, 15.43; Cal, C, 63.16; H, 4.74; N, 15.51; electronic spectra (λ_max_ nm) 440, 510; FT-IR data (KBr, cm^−1^): 1670 ν_(–CH=N)_, 1460 ν_(–N=N–)_; ESI-Mass spectra *m*/*z*: [C_38_H_34_CuN_8_O_4_+H]^+^ = 723.5.

### 3.3. DFT Calculations for Ligand, HL

The whole DFT and TD-DFT measurements were recorded on Gaussian 09 software [41], involving the customary exchange-correlation functional B3LYP (Becke, three-parameter, Lee-Yang-Parr) along with basis set 6-31G(d,p) [42,43].On the basis of optimized ground state geometries, electronic transitions and various optimized structural parameters, such as bond lengths, bond angle, and dihedral angles and hormonal vibrational frequency calculation were determined using same method and basis set. In addition, In addition, few FT-IR vibrations were also shown by animated modes using Gauss View 5 [44]. The parameters such as ∆E, Mulliken electronegativity (χ), dipole moment, chemical potential (μ), global hardness (η), global softness (S), global electrophilicity (ω), absolute softness (σ) and electronic charge (ΔN_max_) were estimated from the DFT results [45]. The Gauge Independent Atomic Orbital (GIAO) method is used in calculating the NMR spectra in CDCl_3_ (using the PCM model) and chemical shifts were measured using corresponding TMS shielding at GIAO-B3LYP/6-311G(d,p) level of theory.

### 3.4. Antimicrobial Assay

The in vitro antimicrobial screening of the ligand and its complexes was studied against the microbes, *Staphylococcus Aureus (Gram positive), Escherichia Coli* (Gram-negative) and *Candida Albicans* and *Aspergillus niger* by Agar well diffusion method [46,47,48,49]. A standard inoculums (10^5^ CFU/mL) of the microbes was spread on the nutrient agar plates using spreader followed by applying wells of 6 mm diameter filled with 100 μL of tested organisms (1 mg m/L). The agar plates containing microbes were incubated for 24–36 h at 37 °C. The antibiotic chloramphenicol was employed as the standard drug against bacterial strains *Staphylococcus Aureus, Escherichia Coli, while* nystatin was used as antifungal reference drug against *Candida Albicans* and *Aspergillus niger* to be used as positive control at 100 μg/mL^–1^ concentration. The susceptibility was determined on the basis of the diameter of the zone of inhibition around the well. All assays were performed at least in duplicate. The dilution test with standard inoculum of 10^5^ cfu mL^−1^ was handled to evaluate the minimal inhibitory concentration (MIC) of the ligand test and its complex. Tests with DMSO were used as a negative control, while chloramphenicol and nystatin as a positive control for bacteria and for fungal strains, respectively, and were performed in parallel. The minimal bactericidal concentration (MBC) was determined by aspirating 0.1 mL of culture medium from each tube on agar plates followed by counting of c.f.u. after 18–24 h of incubation at 35 °C. The results of MIC and MBC are given in Table 3.

## 4. Conclusions

A novel azo-Schiff base ligand and its complexes with Cu(II) ion and Fe(III) ion were reported, and characterized by various physic-chemical methods. All the reported compounds exhibited promising antibacterial activity. However, DFT studies were recorded on ligand and results obtained were comparable with the data existing in literature.

## Figures and Tables

**Table 1:** Quantum chemical parameters.

	Ligand
HOMO	−0.2040 (−5.50 eV)
LUMO	−0.0722 (−1.96 eV)
ΔE	0.1302 (3.54 eV)
Mulliken Electronegativity χ	0.1373
Global Hardness η	0.0651
Absolute softness σ	15.3609
Chemical Potential μ	−0.1373
Global Softness S	7.6804
Global Electrophilicity ω	0.1447
Electronic Charge ΔN	2.1090
Dipole Moment	3.4276

**Table 2:** Selected Optimized geometrical parameters of ligand in the ground state.

Bond Length (Å)	B3LYP/6-31G (d,p)	Ref. [6]	Bond Angles (°)	B3LYP/6-31G (d,p)	Literature
N15-N16	1.232	1.25	N15-N16-C22	119.9	112.2
C19-O27	1.430	1.367	N15-N16-C3	120.0	113.4
C28-N26	1.293	1.304	C19-N20-C21	119.9	120.0
C31-N40	1.343	1.341	C19-N20-C28	120.0	121.2
C31-N26	1.399	1.397	Dihedral angles (°)	B3LYP/6-31G (d,p)	Ref
C3-N15	1.470	1.428	C3-N15-N16-C22	−179.9	−178.1
O27-H29	0.960	0.840	C20-C28-N26-C31	−179.9	--
C20-C21	1.401	1.390	C3-N15-N16-C22	−179.9	--
Bond angles (°)	B3LYP/6-31G (d,p)	Ref. [6]	C21-C20-C19-O27	−179.9	−178.4
C28-N26-C31	119.9	116.6	C28-C20-C19-O27	0.0	−1.1
C31-N40-C32	121.4	118.1	C21-C20-C19-C18	0.0	0.0
C19-O27-H29	109.4	109.5	C28-C20-C19-C18	−179.9	178.4

**Table 3:** Antimicrobial activity of azo-azomethine ligand and its complexes.

Compound Bacterial Strains	Inhibition Zone (mm)					MIC and MBC (μg/mL)			
	Ligand HL	Complex 1	Complex 2	Cam *	DMSO	MIC (MBC) of HL	MIC (MBC) of complex 1	MIC (MBC) of complex 2	MIC (MBC) of Cam
*S. Aureus* (MRSA +ve)	7	13	11	17	No zone	512 (512)	128 (128)	256 (256)	32 (32)
*E. Coli* (Gram −ve)	9	15	17	20	No zone	256 (>256)	64 (256)	256 (>256)	32 (128)
*Fungal strains*	Ligand HL	Complex 1	Complex 2	Nys *		MIC (MBC) of HL	MIC (MBC) of complex 1	MIC (MBC) of complex 2	MIC (MFC) of Nys
*C. Albicans*	No zone	No zone	No zone	22	No zone	ND	ND	ND	50 (50)
*A. Niger*	No zone	No zone	No zone	20	No zone	ND	ND	ND	200 (200)

* Chloramphenicol (Cam) and * Nystatin (Nys) were used as strand drugs for bacteria and fungi, respectively. DMSO was used as a negative control and no effect was detected. ND = Not determined.

## Supplementary Materials

The following are available online, Figure S1: ESI-MS spectrum of ligand, HL.
