# Supplementary material for: Synthesis, Structural Characterization and Antimicrobial Activity of Cu(II) and Fe(III) Complexes Incorporating Azo-Azomethine Ligand
Source: Molecules. 2018 Apr 2;23(4):813. doi: 10.3390/molecules23040813 (PMC6017743; doi:10.3390/molecules23040813)
Supplement: Supplementary file 1 [file molecules-23-00813-s001.pdf]

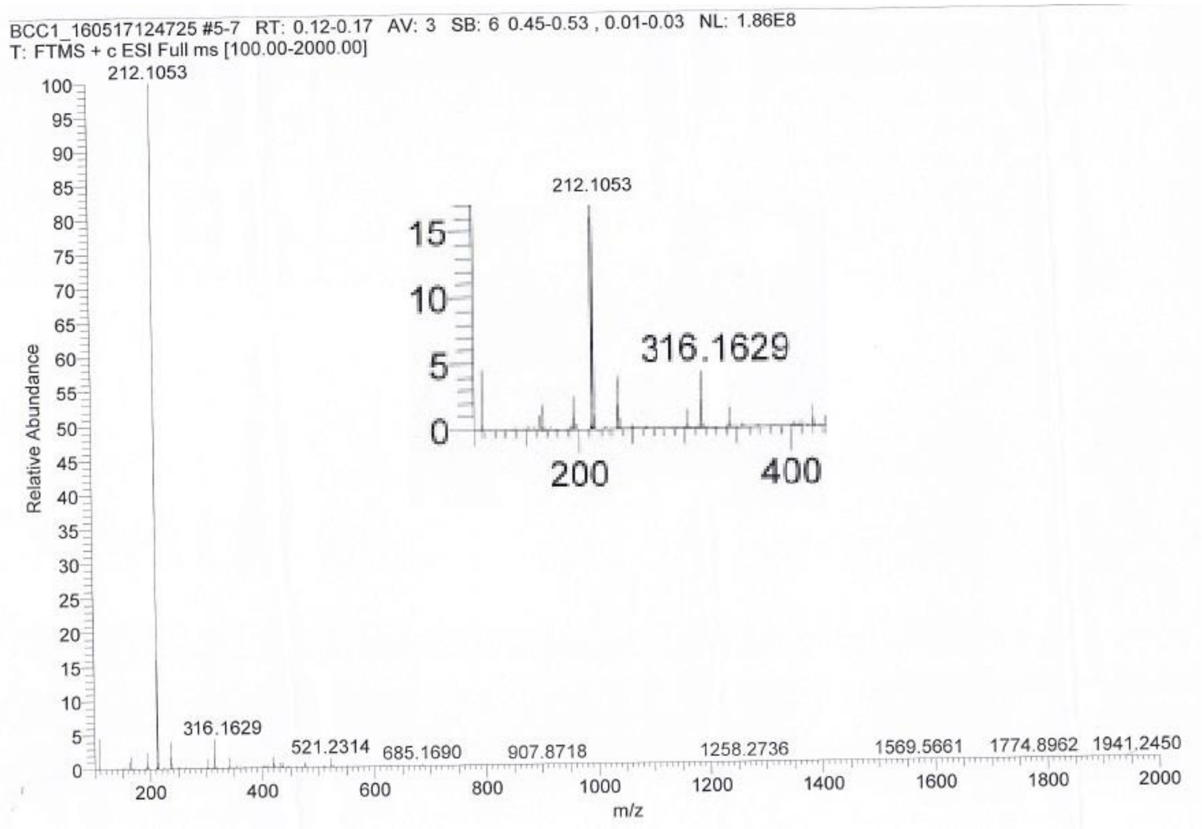

Supplementary Information Figure S1. ESI-MS spectrum of ligand, HL

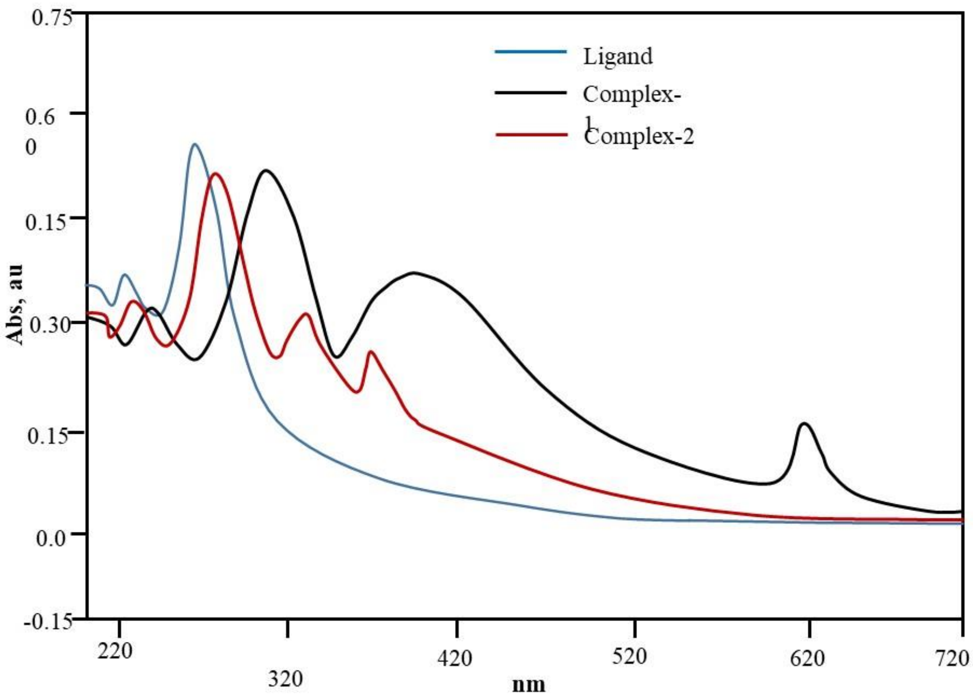

Supplementary Information Figure S2. UV/Vis spectra of ligand and complexes 1 and 2
